# Biological control of *Schistocerca gregaria* and *Locusta migratoria migratorioides* using Entomopathogenic bacteria

**DOI:** 10.1038/s41598-025-87513-7

**Published:** 2025-02-07

**Authors:** Jihan Muhammad, Zeinab Fathy, Saad Moussa

**Affiliations:** 1 Insect Biotechnology and Molecular Biology Unit, Agricultural Research Center 12619, Ministry of Agriculture and Land Reclamation, Plant Protection Research Institute, Giza, Egypt; 2 Department of Pest Physiology, Plant Protection Research Institute, Agricultural Research Center 12619, Ministry of Agriculture and Land Reclamation, Giza, Egypt

**Keywords:** *Photorhabdus luminescens*, *Xenorhabdus nematophila*, *Schistocerca gregaria*, *L. migratoria migratorioides*, Field application, Biological techniques, Microbiology

## Abstract

This study investigated the potential of indigenous entomopathogenic bacterial (EPB) strains from Egypt to control the two most prevalent locust species, *Schistocerca gregaria* (Forsskål) (Orthoptera: Acrididae), and *Locusta migratoria migratorioides* (Reiche & Fairmaire) (Orthoptera: Acrididae). To assess the efficacy of the EPB strains, experiments were conducted in the laboratory, semi field, and field. The results showed that *Xenorhabdus nematophila* (Thomas et Poinar) BA2 (Enterobacterales: Morganellaceae) and *Photorhabdus luminescens* (Thomas et Poinar) EGAP3 (Enterobacterales: Morganellaceae) were the most effective strains against *S. gregaria* and *L. migratoria migratorioides* in laboratory settings. Under semi-field conditions, *X. nematophila* BA2 recorded nymphal mortality rates of 89.31% and 85.00% against the 2^nd^ and 5^th^ nymph instars of *S. gregaria*, respectively, and *P. luminescens* EGAP3 showed nymphal mortality rates of 88.00% and 80.00% against the 2^nd^ and 5^th^ nymph instars of *S. gregaria*, respectively. In field trials, *X. nematophila* BA2 exhibited the highest nymphal mortality rate of 88.70% at 7 days after spraying. Overall, the findings of this study suggest that *X. nematophila* BA2 and *P. luminescens* EGAP3 are promising candidates for environment-friendly, safe locust pest management. Further research is needed to explore and develop these bacteria for commercial use in agriculture.

## Introduction

*Schistocerca gregaria* (Forsskål) (Orthoptera: Acrididae) and *L. migratoria migratorioides* (Reiche & Fairmaire) (Orthoptera: Acrididae) are considered highly damaging pest species in Egypt and other African nations because of their significant economic impact. These notorious pests have inflicted substantial damage to Egypt’s agricultural industry for centuries (The^1,2^). The challenges associated with predicting and managing locust outbreaks are compounded by their recurring nature. The effective management of locust populations requires coordinated efforts involving various stakeholders.

The primary approach to controlling locust populations has traditionally relied on the application of synthetic insecticides, which have proven effective in reducing locust populations in affected areas. However, the use of synthetic pesticides raises concerns regarding potential adverse impacts on both the environment and human health. As an alternative strategy, the utilization of microbial pesticides has emerged as a crucial component of pest management due to their efficacy against a broad range of pests, limited impact on non-target species, and ecological friendliness compared to conventional pesticides^3^.

Locusts and grasshoppers possess a diverse array of natural enemies^4^. However, only a limited number of these have been successfully developed as biological control agents. Prominent examples include the microsporidian *Nosema locustae* ^5,6^ and fungi such as *Metarhizium acridum*^7^, *Aspergillus oryzae* XJ-1^8^, and *Beauveria bassiana*^9^. Additionally, Entomopoxvirus^10^, *Serratia marcescens*^11^, and entomopathogenic nematodes^12^ have shown promise as biocontrol agents.

One highly effective natural technique for insect control involves the use of entomopathogenic nematodes in conjunction with symbiotic bacteria. Infected juveniles of *Heterorhabditis* and *Steinernema* actively search for hosts in the soil, releasing symbiotic bacteria from the genera *Photorhabdus* (Thomas et Poinar) (Enterobacterales: Morganellaceae) and *Xenorhabdus* (Thomas et Poinar) (Enterobacterales: Morganellaceae) into the insect circulatory system^13^. This nematode-bacteria complex serves as an ideal model system for studying both mutualistic and pathogenic symbioses, exhibiting high pathogenicity against a diverse range of insects. The adoption of this strategy has been more prevalent in organic farming than the use of synthetic pesticides, owing to its precise timing and targeted dispersion^14–16^.

*Photorhabdus* and *Xenorhabdus* bacteria produce a wide variety of compounds that contribute to both symbiotic and pathogenic activities^12,17–19^ Notably, even in the absence of nematode hosts, *Photorhabdus* bacteria can be cultured in a medium and still induce insect mortality^20^. These bacteria release numerous secondary metabolites, including lipases, proteases, antibiotics, lipopolysaccharides, and other bioactive substances, into the culture medium^21^. Many of these secondary metabolites generated by these bacteria demonstrate potential applications and have been utilized as biocontrol agents for managing viruses, fungi, nematodes, and insects^22–25^.

In this study, we conducted an assessment of the bioefficacy potential of six entomopathogenic bacterial strains against two species of locusts: the desert locust (*S. gregaria*) and the African migratory locust (*L. migratoria migratorioides*). Furthermore, we examined the impact of these bacterial strains on the activity of carbohydrate-hydrolyzing enzymes in the second and fifth instar. Based on the potential of entomopathogenic bacteria and the limited research on indigenous Egyptian strains, this study aimed to investigate the efficacy of six indigenous Egyptian entomopathogenic bacterial strains against *S. gregaria* and *L. migratoria migratorioides*. Furthermore, the study sought to identify the most promising strains for their potential application as biocontrol agents against locust populations under field conditions.

## Methods

### Bacterial strains and locusts population

We isolated *Photorhabdus luminescens* EGAP1 (region 16S rDNA, GenBank accession number MH368153), *Photorhabdus luminescens* EGAP2 (region 16S rDNA, GenBank accession number MH368154), *Photorhabdus luminescens* EGAP3 (region 16S rDNA, GenBank accession number MH368155), *Photorhabdus luminescens* EGAP4 (region 16S rDNA, GenBank accession number MH368156), *Photorhabdus luminescens* EGAP5 (region 16S rDNA, GenBank accession number MH368157) from their symbiotic entomopathogenic nematode (EPNs) (Supplementary Material 1, Table S1) as described by Ahmed et al.^26^, and *Xenorhabdus nematophila* BA2 obtained from the Pests and Plant Protection Department, National Research Centre, Giza, Egypt. All strains displayed typical Gram-negative morphology: motile, non-spore-forming, non-acid-fast, short rod-shaped bacteria forming small colonies. *Xenorhabdus nematophila* colonies exhibited a polymorphous light brown pigment, while *Photorhabdus luminescens* colonies appeared red. Identification of the entomopathogenic bacteria (EPNs) was confirmed by referring to their colony morphology, physiological, and biochemical characteristics presented in Table 1. Bacterial strains were inoculated on Petri dishes containing Nutrient Agar (NA), bromothymol blue, and 2,3,5-Triphenyltetrazolium chloride (NBTA plates). We ensured the use of bacteria in their primary and active forms based on dye adsorption, pigmentation, and morphology, as described by Han and Ehlers^27^. The bacteria were refreshed weekly on another NBTA plate to ensure their purity and activity for all the experimental trials.

**Table 1:** Characteristics of *P. luminescens* and *X. nematophila.*

Test	*P.lumines-cens* EGAP1	*P.lumines-cens* EGAP2	*P.lumines-cens* EGAP3	*P.lumines-cens* EGAP4	*P.lumines-cens* EGAP5	*X. nema- tophilus BA2*
Colony colour on nutrient agar	Y	Y	R,Y	Y	R	w
Colonycoloron NBTA agar	G	G	G	G	G	R
Colony colour On MacConkey agar	O	O	O	O	O	B
Gram stain	-	-	-	-	-	-
Motility	+	+	+	+	+	+
Bioluminescence	+	+	+	+	+	-
Pathogenicity to						
*G. mellonella*	+	+	+	+	+	+
Lipase activity	+	+	+	+	+	+
Protease activity	+	+	+	+	+	+
Catalase	+	+	+	+	+	-
Oxidase	-	-	-	-	-	-
Glucose	+	+	[ +]^w^	[ +]^w^	[ +]^w^	[ +]^w^
Maltose	+	+	[-]^w^	[-]^w^	[-]^w^	[-]^w^
Lactose	+	-	-	-	-	-

Symbols: B, brown; O, Orange; G, Green; R, red; Y, yellow; P, pink; Pp, pale pink -, Negative; + , positive; the superscript "w" indicates a week reaction.

The locust adults, *L. migratoria migratorioides* and *S. gregaria*, were collected from experimental fields in Atmida village (Dakahlia, Egypt; 30.69° N, 31.80° E) and the El Komy group in El-Farafram (New Valley, Egypt; 25.87° N, 28.38° E), respectively.

Both locusts were cultivated for more than two generations in the gregarious phase within wooden cages measuring 40 × 40 × 30 cm , with a density of 30–60 adults per cage, at the Department of Pest Physiology, Plant Protection Research Institute, Agricultural Research Center (Giza, Egypt; 30.06° N, 31.21° E). To facilitate egg-laying, each cage contained a layer of sand that constituted one-third of its volume. Natural sunlight was used instead of artificial lighting^28^. The cages were cleaned daily, and depending on the season, the locusts were fed fresh alfalfa (*Medicago sativa*) or corn (*Zea mays*) leaves.

### Screening for bioefficacy of EPB strains under laboratory conditions

Microorganism inoculums of 20 μl of pure bacterial stock were placed in 5 ml nutritional broth tubes and cultivated aerobically overnight at 28 °C before being transferred to 50 ml of Luria–Bertani (LB) broth in a 250 ml Erlenmeyer flask, as described previously^25^. Cell densities in the suspensions were calibrated to 4 × 10^7^, 4 × 10^6^, 4 × 10^5^, and 4 × 10^4^ colony-forming units per milliliter (CFU/ml) using a spectrophotometer set at 600 nm. This resulted in a series of bacterial suspensions with varying cell densities, which were then used in the subsequent experiments.

Six strains of EPB were tested in virulence bioassays on the 2^nd^ and 5^th^ nymphal instars of *L. migratoria migratorioides* and *S. gregaria*. All experiments were performed in a completely random order, with 25 treatments that included four different bacterial suspension densities, 4 × 10^4^, 4 × 10^5^, 4 × 10^6^, and 4 × 10^7^ CFU/ml, and a control consisting of the same volume of sterilized LB broth. Separate tests on bacterial suspensions were conducted on nymphs in the 2^nd^ and 5^th^ instars. The tests were repeated three times (three replicates), with 10 nymphs per replicate. The nymphs were individually placed in a glass jar. Koch’s postulates were used to confirm that these bacteria are pathogenic to locusts.

Maize leaves were dipped for 20 s in prepared culture suspensions containing different densities of bacteria. Then, the leaves were air-dried and topically applied once to the nymphs in each treatment group. A similar application of sterilized LB broth was used for the control treatment^29^. Food was changed daily, and the nymphs were kept at 30 °C in an insect-rearing room. The mortality of the nymphs was assessed daily, and observations were recorded for seven days. Cumulative mortality percentages were then calculated using Abbott’s formula (1925)for corrected mortality: Corrected mortality (%) = [(The observed mortality rate in the treatment group—The mortality rate observed in the control group) / (100—The mortality rate observed in the control group)] × 100.

### Determination of lethal concentrations (LC_50_ and LC_90_) of P. luminescens EGAP3 and X. nematophilus BA2

To determine the lethal concentrations (LC_50_ and LC_90_) of the two most lethal strains from the previous experiment (*P. luminescens* EGAP3 and *X. nematophilus* BA2), the previously described methodology was employed. These two strains were then used in all subsequent experiments. Probit analysis was then performed on data from the 2^nd^ and 5^th^ nymphal instars at a significance level of P < 0.05 using LdP Line software^30^.

### Preparation of Samples for biochemical characteristics

Consistent with the previous experiment, 10-s and 10-fifth instar nymphs of *L. migratoria migratorioides* and *S. gregaria* were given contaminated maize leaves that were supplemented with LC_50_ concentrations of bacterial suspensions of *P. luminescens* EGAP3 and *X. nematophilus* (BA2). For the purpose of conducting assays, nymphal samples were collected after 72 h and prepared through homogenization in distilled water, followed by centrifugation at 6000 rpm for 10 min at 5 °C using a Beckman GS-6R centrifuge^25^. The resulting supernatant fluid, which contained the soluble components of the cells, was subsequently aliquoted (0.5 ml) and stored at -20 °C until further analysis. To ensure the accuracy and reliability of the obtained results, three replicates of each biochemical determination were performed.

### Determination of carbohydrate hydrolyzing enzymes activities (amylase, invertase, and trehalase)

Enzyme activities of amylase, invertase, and trehalase were assessed through the digestion of starch, sucrose, and trehalose using a spectrophotometric method. The activities of invertase and amylase were measured according to the protocol established by Ishaaya and Swirski^31^, utilizing the 3,5-dinitrosalicylic acid (DNS) reagent to quantify the free aldehydic groups of glucose released during the digestion of starch or sucrose. Absorbance of the resultant solution was recorded at 540 nm using a spectrophotometer, with glucose concentrations calculated based on a standard glucose curve.

The amylase reaction was composed of 0.1 mL of 2% starch (Sigma, USA), 0.1 mL of 0.2 M phosphate buffer (pH 6.0), and 0.2 mL of a 0.2% enzyme solution. For the invertase reaction, the mixture included 0.2 mL of 4% sucrose (Sigma, USA), 0.1 mL of 0.2 M acetate buffer (pH 5.5), and 0.1 mL of a 0.2% enzyme solution. After incubating the amylase or invertase reaction at 37 °C for 60 min, the enzyme activity was halted by adding 0.8 mL of the DNS reagent (ADVENT CHEMBIO PVT. LTD).The mixture was subsequently heated for 5 min at 100 °C and cooled rapidly in an ice bath. Enzyme activity was then assessed in extinction units (E) at 540 nm using a spectrophotometer (E-Chrom Tech), with results expressed as mg glucose per mg protein per hour.

Trehalase activity was evaluated using a method similar to that of amylase and invertase, employing the DNS reagent to measure glucose produced from trehalose digestion. The reaction mixture for trehalase contained 0.2 mL of 3% trehalose (Sigma, USA), 0.1 mL of 0.2 M acetate buffer (pH 5.5), and 0.1 mL of a 0.2% enzyme extract. To prepare the DNS solution, 1 g of 3,5-dinitrosalicylic acid was dissolved in 20 mL of 2N NaOH and 50 mL of distilled water with the aid of a magnetic stirrer. After adding 30 g of potassium sodium tartrate (Sigma, USA), stirring continued until a clear solution was achieved, and distilled water was added to reach a final volume of 100 mL. All assays were performed in duplicate, with each assay repeated at least three times.

### Assessment of the effectiveness of P. luminescens EGAP3 and X. nematophilus BA2 in semi-field trials

The semi-field experiments were conducted at the Plant Protection Institute (Giza, Egypt, 30.06° N, 31.21° E) during May and June 2021. In these experiments, nymphs of *S. gregaria*, specifically the 2^nd^ and 5^th^ instar, were used. Plastic pots with dimensions of 16 cm (upper diameter) and 20 cm (height) were filled with clay soil. The pots were then planted with corn (*Zea mays*) and treated with bacterial suspensions of *P. luminescens* EGAP3 and *X. nematophilus* BA2. The concentrations of these bacterial suspensions were 2.10 × 10^5^ and 1.62 × 10^5^ CFU/ml (LC_50_) and 4.14 × 10^7^ and 3.11 × 10^7^ CFU/ml (LC_90_) for 2^nd^ instar, and 4.12 × 10^5^ and 2.71 × 10^5^ CFU/ml (LC_50_) and 2.63 × 10^8^ and 1.51 × 10^8^ CFU/ml (LC_90_) for 5^th^ instar, respectively. As a control, the plants were treated with sterile distilled water. Each treatment involved the release of 20 nymphs. To prevent nymphs from escaping and natural enemies from entering, the pots were covered with a fine mesh fabric of tulle. Optimal environmental conditions were maintained by placing all pots on water-filled trays at a height of 1 m on a countertop. The experimental design included five replicates for each treatment, with distinct bacterial strains, and the entire process was repeated twice to ensure the reliability of the results. Nymph mortality was assessed daily for a period of seven days, following the methodology described in the study by Wakil et al.^32^. Deceased nymphs and their remains were collected daily to prevent scavenging by live locusts.

### Fecundity and fertility assays

In the previous experiment, fifth instars were exposed to the LC_50_ concentrations of both *P. luminescens* EGAP3 and *X. nematophilus* (BA2) after reaching adulthood. Male and female nymphs (15 couples) were sexed and immediately paired in individual 2-L plastic boxes containing food. These boxes were placed under the same conditions used for mass rearing. The following parameters were recorded for both the control and treated insects: fecundity (number of eggs deposited by the female during its lifespan), fertility (the percentage of hatched eggs relative to eggs laid per female)^33^. The eggs were incubated in a substratum composed of two-thirds peat and one-third sand at a temperature of 33 °C.

### Field experiments

During the 2022 season, the following studies were conducted in two specific fields. The first field, located in Atmida village (Dakahlia, Egypt; 30.69° N, 31.80° E), experienced infestations of African locusts and grasshoppers. The locust infestation was predominantly composed of 2^nd^ and 3^rd^ nymphal instars. Population densities per square meter before treatment varied across species: *L. migratoria migratorioides* (2–3 individuals), *Eyprepocnemis plorans* (Charpentier) (Orthoptera: Acrididae) (8–9 individuals), *Heteracris annulosa* (Walker) (Orthoptera: Acrididae) (1–3 individuals), and *Acrotylus insubricus* (Scopoli) (Orthoptera: Acrididae) (1–2 individuals). The crop cultivated in this field was maize (*Zea mays*).

The second field, known as the El Komy group in El-Farafram (New Valley, Egypt; 25.87° N, 28.38° E), was infested with desert locusts. The infestation primarily involved nymphal instar individuals ranging from 2^nd^ to 5^th^ instars. The reported population density of desert locusts in this field ranged from 15 to 20 nymphs per square meter. The crop cultivated in this field was basil (*Ocimum basalicum*).

### Assessment of the bioefficacy of P. luminescens EGAP3 and X. nematophilus BA2 in field trials

The most effective strains, *P. luminescens* EGAP3 and *X. nematophilus* BA2, were used to evaluate bioefficacy under field conditions. Each experiment consisted of 18 plots (20 × 30 m^2^). The plots were isolated by a wide belt of 10 × 30 m^2^ to prevent immigration of treated nymphs to the other plots and control. Three treatments (six plots each) were established: *P. luminescens* EGAP3, *X. nematophilus* BA2 (diluted 40 g of cells with 200 times the volume of clean water before spraying, for a concentration of 4 × 10^8^ cells/mL), and a control (untreated). The treatments were applied using a 20-L Cifarelli motorized knapsack mist blower sprayer at a rate of 20 L per plot. The flow rates of the spray solution and spray swath width were measured using the method described by Cressman and Dobson^34^. The nymphal population was recorded (live/dead) before spray treatment and then at 3, 5, and 7 days. *P. luminescens* and *X. nematophilus* were isolated from dead nymphs to verify the presence of bacteria. A non-uniform population of nymphs was observed, so Sun-Shepard’s formula was used to correct for control mortality^35^: Corrected mortality (%) = [(mortality (%) in treated plot—change (%) in control plot population)/100—change (%) in control plot population)] × 100. Where change (%) is the percent variation compared to the control.

### Statistical analysis

The percentage data underwent arcsine transformation prior to statistical analysis to meet the assumptions of normality and homogeneity of variance. Analysis of variance (ANOVA) was then conducted to assess the effects of the treatments on the experimental data obtained from the laboratory, semi-field experiments and field expermients. Following ANOVA, treatment means were compared using Tukey’s HSD test at a significance level of α = 0.05. Statistical analyses were performed using COSTAT software (version 6.45).

## Results

### Screening for bioefficacy of EPB strains under laboratory conditions

The laboratory study revealed that second and fifth instar of the desert locust (*S. gregaria*) and the migratory locust (*L. migratoria migratorioides*) exhibited susceptibility to all tested strains of entomopathogenic bacteria (EPB) after 7 days of exposure. Furthermore, the results revealed significant differences in mortality rates between several pairs of bacterial strains (Table 2).

**Table 2:** Effect of six entomopathogenic bacterial (EPB) strains on nymphal mortality under laboratory conditions after 7 days of treatment.

Treatments	Corrected nymphal mortality percentages (mean ± SE) over control (4 × 10^7^)			
	*S. gregaria*		*L. migratoria migratorioides*	
	2^nd^ instar	5^th^ instar	2^nd^ instar	5^th^ instar
*P. luminescens* EGAP1	50.00 ± 5.77^c^	43.33 ± 6.81^c^	46.66 ± 6.66^c^	40.00 ± 5.77^c^
*P. luminescens* EGAP2	63.33 ± 1.85^b^	50.00 ± 5.77^c^	63.33 ± 3.33^b^	46.66 ± 3.60^b^
*P. luminescens* EGAP3	90.00 ± 5.50^a^	83.33 ± 3.33^a^	87.66 ± 3.38^a^	80.73 ± 5.81^a^
*P. luminescens* EGAP4	70.00 ± 5.77^b^	56.66 ± 2.31^c^	66.66 ± 4.50^b^	46.66 ± 3.71^b^
*P. luminescens* EGAP5	73.33 ± 4.40^b^	63.31 ± 6.66^b^	70.00 ± 5.77^b^	53.31 ± 2.40^b^
*X. nematophila *BA2	91.00 ± 4.93^a^	88.66 ± 4.63^a^	89.31 ± 5.20^a^	86.66 ± 3.38^a^
Water spray	8.31 ± 0.57	10.33 ± 0.88	9.66 ± 0.66	14.33 ± 0.84
F-values	5.60	10.37	8.33	19.30
df	5, 12	5, 12	5, 12	5, 12
P-value	= 0.0068	= 0.0005	= 0.0013	< 0.001

Analyzed using a one-way analysis of variance (ANOVA). Different letters within each column indicate significant differences in nymphal mortality rates between EPB strains (Tukey’s HSD, p < 0.05).

The comparison of virulence among six entomopathogenic bacterial (EPB) strains revealed that *P. luminescens* EGAP3 and *X. nematophila* BA2 caused the highest mortality for the second and fifth nymphal instars of *S. gregaria* and *L. migratoria migratorioides*. Notably, *X. nematophila* BA2, the strain with the highest overall virulence, also demonstrated the lowest LC_50_ and LC_90_ values for both locust species at both nymphal stages (Table 3).

**Table 3:** lethal activities LC_50_ and LC_90_ CFU/ml − 1 of *P. luminescens* EGAP3 and *X. nematophila* BA2.

Treatment	S. gregaria								
	Locusinstar	LC_50_ CFU/ml	Lower limit	Upper limit	LC_90_ CFU/ml	Lower limit	Upper limit	Slope ± S.E	Chi-square (χ^2^)
P. luminescens EGAP3	2^nd^	2.10 × 10^5^	1.42 × 10^5^	3.91 × 10^5^	4.14 × 10^7^	2.40 × 10^7^	8.42 × 10^7^	0.56 ± 0.04	0.26
	5^th^	4.12 × 10^5^	2.73 × 10^5^	6.10 × 10^5^	2.63 × 10^8^	1.11 × 10^8^	7.57 × 10^8^	0.46 ± 0.03	0.55
X. nematophila (BA2)	2^nd^	1.62 × 10^5^	1.00 × 10^5^	2.33 × 10^5^	3.11 × 10^7^	1.81 × 10^7^	6.00 × 10^7^	0.56 ± 0.04	0.04
	5^th^	2.71 × 10^5^	1.69 × 10^5^	4.00 × 10^5^	1.51 × 10^8^	7.00 × 10^7^	3.90 × 10^8^	0.47 ± 0.04	2.97
L. migratoria migratorioides									
P. luminescens (EGAP3)	2^nd^	2.91 × 10^5^	1.94 × 10^5^	4.13 × 10^5^	7.63 × 10^7^	4.00 × 10^7^	1.59 × 10^8^	0.53 ± 0.07	1.13
	5^th^	5.90 × 10^5^	3.91 × 10^5^	8.66 × 10^5^	4.00 × 10^8^	1.73 × 10^8^	1.20 × 10^9^	0.45 ± 0.03	1.10
X. nematophila (BA2)	2^nd^	2.11 × 10^5^	1.40 × 10^5^	3.00 × 10^5^	4.12 × 10^7^	2.44 × 10^7^	8.34 × 10^7^	0.56 ± 0.04	0.21
	5^th^	3.72 × 10^5^	2.31 × 10^5^	5.51 × 10^5^	2.44 × 10^8^	1.11 × 10^8^	7.33 × 10^8^	0.45 ± 0.03	0.19

Lower and upper limits represent the 95% confidence interval of the estimated LC_50_ and LC_90_ values.

### Determination of carbohydrates hydrolyzing enzymes activities (amylase, invertase, and trehalase)

**Fig.1:**
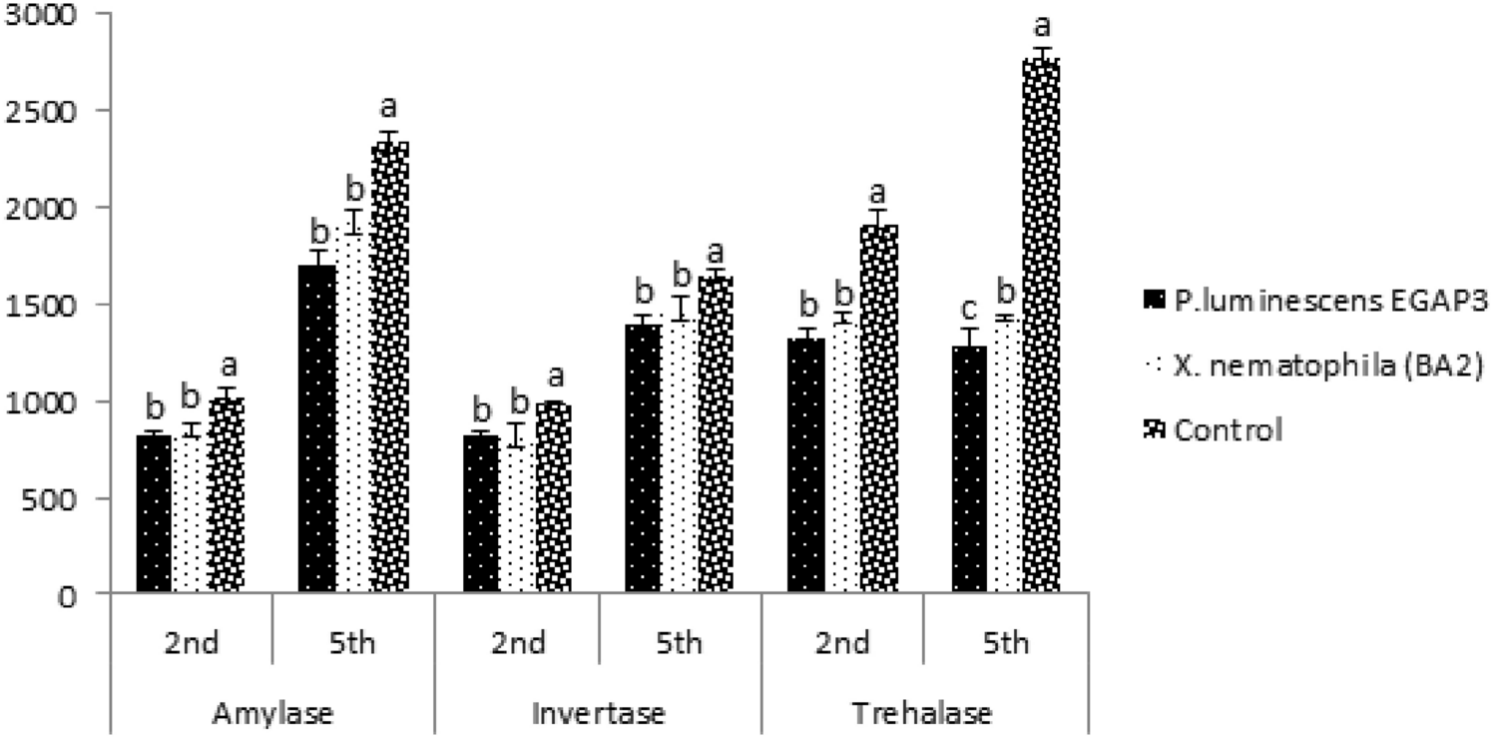
Effect of *P. luminescens* EGAP3 and *X. nematophila* BA2 on amylase, invertase, and trehalase enzymatic activities of *S. gregaria.* Different letters (**a**, **b**) indicate significant differences (p < 0.05) as measured with Tukey’s HSD test.

The results of the carbohydrates hydrolyzing enzymes in the 2^nd^ and 5^th^ instars of *S. gregaria* and *L. migratoria migratorioides* treated with the LC_50_ concentration of *P. luminescens* EGAP3 and *X. nematophila* BA2 revealed a statistically significant decrease compared to the controls. The data in Fig. 1 showed significant (P < 0.05) differences in the levels of amylase (2^nd^ instar: df = 2, F = 103.10, P < 0.001; 5^th^ instar: df = 2, F = 7.40, P = 0.0240), invertase (2^nd^ instar: df = 2, F = 40.35, P < 0.001; 5^th^ instar: df = 2, F = 28.74, P < 0.001), and trehalase (2^nd^ instar: df = 2, F = 122.73, P < 0.001; 5^th^ instar: df = 2, F = 3220.82, P < 0.001) in *S. gregaria* as determined by one-way ANOVA.

**Fig. 2:**
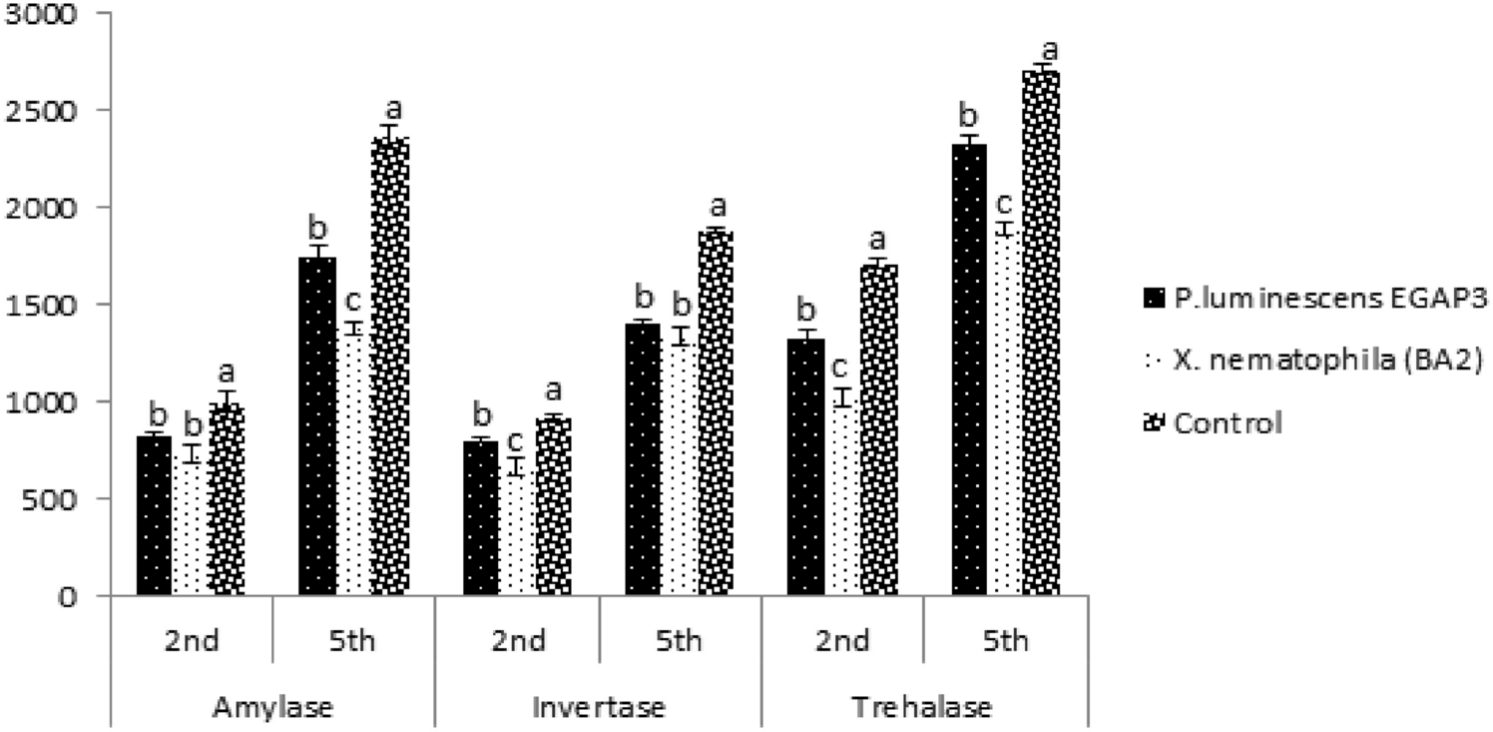
Effect of *P. luminescens* EGAP3 and *X. nematophila* BA2 on amylase, invertase, and trehalase enzymatic activities of *L. migratoria migratorioides.* Different letters (**a**, **b**) indicate significant differences (p < 0.05) as measured with Tukey’s HSD test.

Similarly, in *L. migratoria migratorioides* (Fig. 2), significant alterations were observed in amylase (2^nd^ instar: df = 2, F = 13.71, P = 0.0058; 5^th^ instar: df = 2, F = 97.05, P < 0.001), invertase (2^nd^ instar: df = 2, F = 18.56, P = 0.0027; 5^th^ instar: df = 2, F = 113.65, P < 0.001), and trehalase (2^nd^ instar: df = 2, F = 70.55, P < 0.001; 5^th^ instar: df = 2, F = 113.85, P < 0.001).

### Assessment of the effectiveness of P. luminescens EGAP3 and X. nematophilus BA2 in semi-field trials

The corrected mortality percentages resulting from the treatments with LC_50_ and LC_90_ concentrations of *P. luminescens* EGAP3 and *X. nematophila* BA2 were determined using^36^ formula Seven-day treatment data showed no significant effects for either bacterial strain (Table 4). The findings demonstrated that insecticidal fluids containing higher LC_90_ concentrations of *X. nematophila* BA2 exhibited high efficacy against 2^nd^ and 5^th^ instars, respectively. In contrast, the LC_50_ concentrations of *P. luminescens* EGAP3 were found to be less effective against 2^nd^ and 5^th^ instars, respectively.

**Table 4:** Effect of *P. luminescens* EGAP3 and *X. nematophila* BA2 on *S. gregaria* nymphal mortality under semi field conditions after 7 days of treatment.

Treatments	Corrected nymphal mortality percentages (mean ± SE) over control (1 × 10^8^)			
	LC_50_ CFU/ml		LC_90_ CFU/ml	
	2^nd^ instars	5^th^ instars	2^nd^ instars	5^th^ instars
*P. luminescens* EGAP3	48.00 ± 2.08^a^	47.00 ± 2.08^a^	88.00 ± 1.15^a^	80.00 ± 1.15^a^
*X. nematophila *BA2	49.00 ± 2.64^a^	47.71 ± 2.02^a^	89.31 ± 1.76^a^	85.00 ± 2.30^a^
Water spray	8.71 ± 0.20	9.33 ± 0.51	10.44 ± 0.40	11.31 ± 0.91
F-values	0.08	0.05	0.44	3.20
df	1, 4	1, 4	1, 4	1, 4
P-value	= 0.7815	= 0.8298	= 0.5408	= 0.1478

Analyzed using a one-way analysis of variance (ANOVA). Different letters within each column indicate significant differences in nymphal mortality rates between EPB strains (Tukey’s HSD, p < 0.05).

### Fecundity and fertility assays

The results obtained from the study demonstrate that the bacterial strains *P. luminescens* EGAP3 and *X. nematophila* (BA2) have a significant negative impact on the reproductive potential and ovarian development of *S. gregaria* adults that emerged from 5^th^ instar treated with the LC_50_ concentrations of both bacterial strains (Table 5). Fecundity, fertility, and overall egg production were notably reduced in the treated groups, indicating the adverse effects of the treatment. Furthermore, the treatment disrupted the normal pattern of egg deposition by inducing delays in ovarian maturation and affecting the egg-laying cycle.

**Table 5:** Effect of LC_50_ of *P. luminescens* EGAP3 and *X. nematophila* BA2 on the fecundity, fertility of adult female desert locust *S. gregaria* at 25 ± 2 °C and 60% RH:

	Fecundity ± SE	Fertility ± SE	Ne/Ep ± SE
Control	135.48 ± 8.33^a^	96.00 ± 3.44^a^	55.33 ± 2.33^a^
*P. luminescens* EGAP3	94.50 ± 3.01^b^	75.72 ± 2.32^b^	35.00 ± 2.01^b^
*X. nematophila* BA2	72.40 ± 4.34^c^	47.33 ± 4.31^c^	23.81 ± 3.02^c^
F-values	57.50	34.54	44.84
df	2, 6	2, 6	2, 6
P-value	< 0.001	< 0.001	< 0.001

Analyzed using a one-way analysis of variance (ANOVA). The letters (a, b, c) indicate significant differences between the control and treatment groups, respectively, at P < 0.05, fecundity (number of eggs deposited by the female during its lifespan), fertility (number of hatched eggs / number of eggs laid per female) × 100%), Ne/Ep, number of eggs by egg-pod.

### Assessment of the bioefficacy of P. luminescens EGAP3 and X. nematophilus BA2 in field trials

The field efficacy of the selected EPB strains, which had previously demonstrated the highest mortality rates in experiments, was examined. Under field conditions, no significant differences in mortality rates were observed between the selected EPB strains across both fields (Table 6). The application of the selected EPB resulted in a pooled nymphal mortality rate ranging from 80.74% to 88.70% after 7 days, with *X. nematophila* BA2 yielding the highest mortality rate.

**Table 6:** Effect of *P. luminescens* EGAP3 and *X. nematophila* BA2 on nymphal mortality under field conditions.

Treatments	Corrected nymphal mortality percentages (mean ± SE) over control (1 × 10^8^)							
	*L. migratoria migratorioides* and grasshoppers controled in Atmida village field				*S. gregaria* controled in El Komy group field			
	3 days		5 days	7 days	3 days		5 days	7 days
*P. luminescens* EGAP3	40.00 ± 2.80^a^		74.88 ± 2.33^a^	80.74 ± 2.91^a^	54.30 ± 2.30^a^		81.11 ± 4.81^a^	85.33 ± 2.60^a^
*X. nematophila* BA2	34.70 ± 2.91^a^		65.35 ± 1.83^a^	81.60 ± 4.58^a^	39.61 ± 2.10^b^		70.42 ± 3.51^a^	88.70 ± 3.10^a^
Water spray	8.48 ± 0.20		10.70 ± 1.22	11.72 ± 0.50	10.40 ± 0.22		9.70 ± 0.33	8.90 ± 0.71
F-values	4.60		6.00	0.12	22.87		4.59	0.58
df	1, 4		1, 4	1, 4	1, 4		1, 4	1, 4
P-value	= 0.0647		= 0.0705	= 0.7449	= 0.0088		= 0.0987	= 0.4859

Analyzed using a one-way analysis of variance (ANOVA). Different letters within each column indicate significant differences in nymphal mortality rates between EPB strains (Tukey’s HSD, p < 0.05).

**Fig.3:**
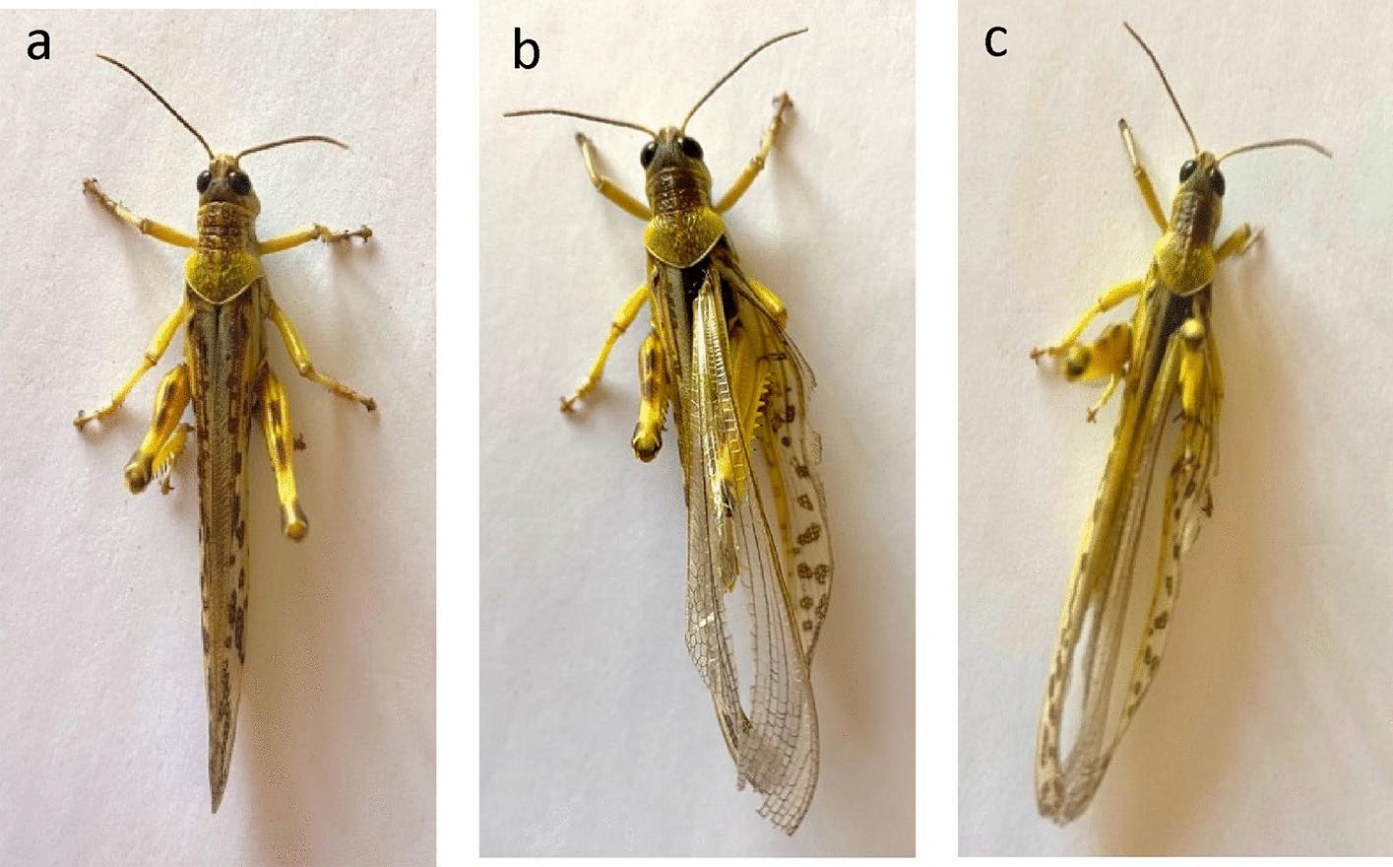
(**a**) Control adult of *S. gregaria* (**b**) Malformed adult that emerged from 5^th^ instar that treated with *P. luminescence* EGAP3. (**c**) Malformed adult that emerged from 5^th^ instar that treated with *X. nematophila* BA2.

**Fig.4:**
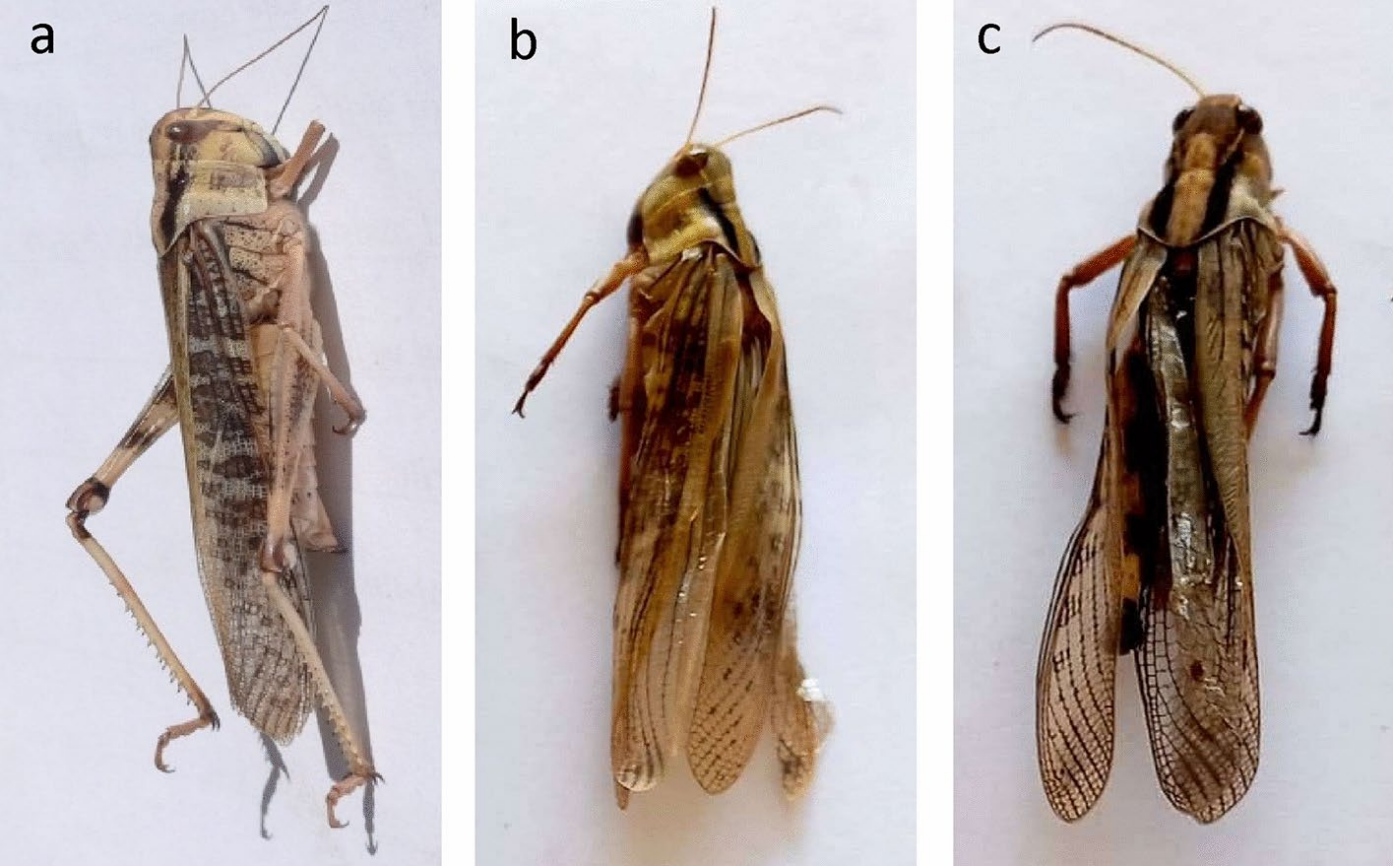
(**a**) Control adult of *L. migratoria migratorioides* (**b**) Malformed adult that emerged from 5^th^ instar that treated with *P. luminescence* EGAP3. (**c**) Malformed adult that emerged from 5^th^ instar that treated with *X. nematophila* BA2.

Furthermore, the emergence of deformities in adult individuals of both *S. gregaria* (Fig. 3 and *L. migratoria migratorioides* (Fig. 4) was observed. These deformities were observed specifically among individuals that had undergone development from 5^th^ instar and were subjected to treatment with *P. luminescens* EGAP3 and *X. nematophila* BA2.

## Discussion

The results obtained from laboratory, semi-field, and field trials conducted in this study provide evidence that *P. luminescens* EGAP3 and *X. nematophila* BA2 significantly contribute to the mortality of *L. migratoria migratorioides* and *S. gregaria*. These findings are consistent with the research conducted by Fathy et al.^37^ who reported higher mortality rates in second and fifth-instar *S. gregaria* nymphs as a result of the potent toxicity exhibited by *P. luminescens* bacterial suspension and its cell-free filtrate in laboratory trials. Furthermore, the results align with the findings of Fathy et al.^12^ which demonstrated the lethal effects of entomopathogenic nematodes *Steinernema* spp. and *H. bacteriophora* (HP88), along with their mutualistic bacteria *Xenorhabdus* spp and *P. luminescens*, on fifth-instar and adult stages of the African migratory locust *Locusta migratoria* in semi-field trials.

Similarly, the results are in accordance with the research conducted by Muhammad et al.^25^, which highlighted the effectiveness of *P. luminescens* against the second and fifth instars of the migratory locust, *L. migratoria migratorioides*. Additionally, these findings support the observations made by Souad et al.^38^, who demonstrated the high susceptibility of locusts to entomopathogenic nematodes along with their mutualistic bacteria, *P. luminescens*, in a dose- and time-dependent manner, resulting in mortality rates of up to 80% after 120 h.

Our findings align with previous research by Gerdes et al.^39^ who documented the insecticidal properties of *Photorhabdus luminescens*. This convergence of evidence suggests the potential application of *P. luminescens* as a biopesticide. Employing a single, naturally derived product for pest control offers a potentially more sustainable and environmentally friendly alternative to the current reliance on a diverse range of synthetic chemical pesticides.

Symbiotic entomopathogenic nematode (EPN) bacteria, such as *P. luminescens* and *Xenorhabdus*, are widely acknowledged for their production of various substances that serve important physiological functions^23,40^. These bacteria, along with the nematodes, play a vital role in killing and decomposing the insect host, which serves as a source of nutrition for both bacteria and nematodes. One of the mechanisms through which these bacteria influence insects is by modulating the activity of carbohydrate-hydrolyzing enzymes. These enzymes are responsible for breaking down complex carbohydrates into simpler sugars that insects can absorb. Carbohydrate-hydrolyzing enzymes are produced by the salivary glands and midgut epithelium, and they are essential for insect development and survival.

Our findings align with the research conducted by Fathy et al.^12^, who demonstrated a significant reduction in the levels of protease, amylase, invertase, and trehalase—digestive enzymes involved in carbohydrate hydrolysis—in both fifth-instar and adults of the African migratory locust, *L. migratoria migratorioides*, following exposure to *Steinernema* sp. (SII) and *H. bacteriophora* (HP88). Furthermore, our results are consistent with the findings ofMuhammad et al.^25^, who emphasized the capacity of *Photorhabdus* to produce orally active insecticidal toxins that target locusts.

The findings of our study provide evidence that bacterial cultures can reduce the reproductive activities of desert locusts, including fecundity and fertility. Moreover, our research indicates that the effectiveness of these reproductive deterrents varies depending on the bacterial species employed. In particular, *X. nematophila* BA2 demonstrated superior reproductive deterrent activities against the treated locusts compared to *P. luminescens* EGAP3.

These results align with recent research conducted by Vicente-Díez et al.^41^, which investigated the impact of deterrent compounds produced by *Xenorhabdus nematophila* and *Photorhabdus laumondii* on the ovipositional behavior of grapevine moths (*Lobesia botrana*). Vicente-Díez et al.^41^. found that the effect of these deterrent compounds varied based on the bacterial species, the use of bacterial cell-free supernatants or unfiltered fermentation products, and the duration of the culture. For instance, soaking grapes in 3-day ferments of *X. nematophila* and *P. laumondii* led to a reduction of approximately 55% and 95% in the number of eggs laid, respectively, compared to the control.

The results obtained from our study demonstrated that *P. luminescens* EGAP3 and *X. nematophila* BA2 exhibited satisfactory mortality rates against the second and fifth nymph instars of locusts within a period of three to seven days, both in laboratory and semi-field conditions. However, when evaluated under field conditions, these bacterial products were found to reduce the number of locust instars within a seven-day period. Moreover, these bacteria have the capability to inhibit carbohydrate-hydrolyzing enzymes in the insect gut, leading to disruption in nutrient acquisition and subsequent locust mortality. This convergence of evidence highlights the potential for using a single, naturally derived product for pest control, offering a sustainable and environmentally friendly alternative to synthetic chemical pesticides. It is important to conduct further investigations to assess the potential long-term effects of these bacteria on non-target organisms and the environment. Based on these findings, it can be inferred that *P. luminescens* EGAP3 and *X. nematophila* BA2 hold promise as effective candidates for the biological control of locusts. Also, future research is essential to explore the practical applications of these bacteria in managing acridid populations. Future research should investigate the feasibility of cultivating these bacteria in sufficient quantities for large-scale treatments. Understanding how to optimize their production and application will be critical for integrating biocontrol methods into existing pest management strategies.

## In conclusion

Traditionally, locust control has been dominated by the application of synthetic insecticides, demonstrably effective in curbing populations. However, concerns regarding potential environmental and human health ramifications associated with synthetic pesticide use necessitate the exploration of alternative control methods. This study explored the efficacy of six entomopathogenic bacteria (EPB) strains in controlling desert locust (*S. gregaria*) and migratory locust (*L. migratoria migratorioides*). Laboratory, semi-field, and field trials evaluated the bacteria’s impact. All tested EPB strains displayed locust mortality within 7 days under laboratory conditions. *P. luminescens* EGAP3 and *X. nematophila* BA2 emerged as the most virulent, causing significant mortality across various nymphal stages in both locust species. The study further investigated the effect of these two potent strains on locust nymphs’ carbohydrate-hydrolyzing enzyme activities. Enzyme activity significantly decreased compared to controls, suggesting a potential mechanism for their effectiveness. Semi-field trials revealed that higher concentrations of X. nematophila BA2 were more successful against locust nymphs, while *P. luminescens* EGAP3 showed lower efficacy. For both bacterial strains, assessments of fecundity and fertility in adult locusts that emerged from treated nymphs indicated negative effects on reproduction. Treated groups displayed reduced egg production and disruptions in ovarian development. Field trials corroborated the efficacy of *P. luminescens* EGAP3 and *X. nematophila* BA2, with *X. nematophila* BA2 causing the highest mortality rate. Additionally, adult locusts from treated nymphs exhibited deformities in both species.

These findings align with previous research on the effectiveness of *P. luminescens* and *Xenorhabdus* spp. against locusts and other insect pests^25,37,39,41^. The study highlights the potential of *P. luminescens* EGAP3 and *X. nematophila* BA2 as biopesticides for locust control. However, further research is required to assess their long-term impact on non-target organisms and the environment. Overall, the study demonstrates the promise of these two EPB strains as biological control agents for locust populations.

## Supplementary Information

Supplementary Information.

## Data Availability

The data that support the findings of this study are available from the corresponding author, upon reasonable request.
