# Supplementary material for: Biological control of Schistocerca gregaria and Locusta migratoria migratorioides using Entomopathogenic bacteria
Source: Sci Rep. 2025 Feb 7;15:4601. doi: 10.1038/s41598-025-87513-7 (PMC11806088; doi:10.1038/s41598-025-87513-7)
Supplement: Supplementary file 1 — Supplementary Information. [file 41598_2025_87513_MOESM1_ESM.docx]

# Supplementary Material

# Table S1. Source of entomopathogenic nematodes

| **Nematodes Species** | **Population** | **Geographic**  **location** | **Crop** | **Source*** |
| --- | --- | --- | --- | --- |
| *Heterorhabdities indica* | EGAZ1 | Sues, Egypt | Nktarin | Soil |
| *Heterorhabdities indica* | EGAZ2 | El-Kasasein, Ismailia, Egypt | Mango | Soil |
| *Heterorhabdities indica* | EGAZ3 | El-Kasasein, Ismailia, Egypt | Palm | Soil |
| *Heterorhabdities indica* | EGAZ4 | Sues, Egypt | Plum | Soil |
| *Heterorhabdities indica* | EGAZ5 | El-Kasasein, Ismailia, Egypt | Alfalfa | Soil |
| *Heterorhabdities bacteriophora* | HP88 | USA | Commercial product | BioLogic, Inc., USA |
| *Steinernema carpocapsae* | All | USA | Commercial product | BioLogic, Inc., USA |

Nematodes were isolated from soil using last instar larvae of *Galleria mellonella* as bait.
